# Coinfections with SARS-CoV-2 and other respiratory pathogens

**DOI:** 10.1017/ice.2020.322

**Published:** 2020-07-03

**Authors:** Aniruddha Hazra, Maggie Collison, Jennifer Pisano, Madan Kumar, Cassandra Oehler, Jessica P. Ridgway

**Affiliations:** University of Chicago Medicine, Chicago, Illinois

Although initial data from China found coinfection between severe acute respiratory syndrome coronavirus 2 (SARS-CoV-2) and other respiratory pathogens to be rare, a report from the United States suggests this to be significantly more common.^1,2^ Because many hospitals utilize the presence of other respiratory infections to stratify risk among patients under investigation (PUI) for coronavirus disease 2019 (COVID-19), better understanding of coinfections is critical. In this brief report, we describe coinfections between SARS-CoV-2 and other respiratory pathogens at a large urban medical center in Chicago, Illinois.

## Methods

In March 2020, our laboratory (University of Chicago Medicine) began in-house real-time reverse transcriptase–polymerase chain reaction (RT-PCR) testing for SARS-CoV-2 from nasopharyngeal (NP) swabs (Roche cobas SARS-CoV-2 RT-PCR assay, Xpert Xpress SARS-CoV-2 test). Notably, no cross reactivity with non–SARS-CoV2 coronaviruses has been observed with these assays because primer design is specific to the SARS-CoV-2 genome.^3^ The same specimen can be tested via RT-PCR for a respiratory panel (RP) of other common pathogens, including adenovirus, coronavirus 229E/HKU1/NL63/OC43, human metapneumovirus, influenza A/B, parainfluenza 1–4, respiratory syncytial virus, *Mycoplasma pneumoniae*, *Chlamydophila pneumoniae*, *Bordetella pertussis*, and rhinovirus/enterovirus (BioFire FilmArray respiratory panel 2). This report examines patients with influenza-like illness symptoms who were simultaneously tested for SARS-CoV-2 and the above panel from March 12, 2020, through April 15, 2020.

We stratified the specimens by SARS-CoV-2 positivity and compared those that tested positive for a RP pathogen in each group with the χ^2^ test and the Fisher exact test. We also calculated the median age in each subgroup and compared them using a 2-sample *t* test with equal variances. All statistical testing used a *P* < .05 level of statistical significance. We used STATA version 15 software (StataCorp, College Station, TX) for all analyses.

## Results

During the observed period, 2,535 specimens were simultaneously tested for SARS-CoV-2 and RP pathogens on 2,458 symptomatic patients. The overwhelming majority of tests (1,214 of 2,535, 47.9%) were collected in the emergency department or from inpatients (1,042 of 2,535, 40.1%). Overall, 459 (18.1%) were positive for SARS-CoV-2 and 364 (14.4%) were positive for at least 1 RP pathogen. The most common RP pathogens found were rhinovirus–enterovirus (7.1%), influenza A (2.1%), coronavirus NL63 (2.1%), and human metapneumovirus (2.0%).

Of the specimens positive for SARS-CoV-2, 15 (3.3%) were also positive for at least 1 RP pathogen (Table 1). Patients coinfected with SARS-CoV-2 and a RP pathogen (median, 39 years) were significantly younger than those with only SARS-CoV-2 infection (median, 58 years; 95% confidence interval [CI], 2.2–20.7; *P* = .02). These coinfections were most common with rhinovirus–enterovirus (8 of 15, 53%). Of those negative for SARS-CoV-2, 349 (16.8%) were positive for at least 1 RP pathogen and 33 (1.8%) were positive for 2 or more RP pathogens. Detection of any RP pathogen was significantly lower in specimens positive for SARS-CoV-2; coronavirus NL63, human metapneumovirus, influenza A, respiratory syncytial virus, and rhinovirus/enterovirus occurred significantly less frequently in specimens positive for SARS-CoV-2.

**Table 1.** Positivity of Respiratory Panel Pathogens Stratified by SARS-CoV-2 Status

Panel Pathogen	SARS-CoV-2 Positive, No. (%) (n = 459)	SARS-CoV-2 Negative, No. (%) (n = 2,076)	*P* Value^a^
Respiratory panel	15 (3.3)	349 (16.8)	**<.001**
Adenovirus	2 (0.4)	25 (1.2)	
Coronavirus 229E	0 (0.0)	7 (0.3)	
Coronavirus HKU1	0 (0.0)	13 (0.5)	
Coronavirus NL63	1 (0.2)	51 (2.5)	**.002**
Coronavirus OC43	0 (0.0)	10 (0.5)	
Human metapneumovirus	2 (0.4)	48 (2.3)	**.009**
Influenza A	3 (0.7)	49 (2.4)	**.020**
Influenza B	0 (0.0)	13 (0.6)	
Parainfluenza 1	0 (0.0)	1 (0.0)	
Parainfluenza 2	1 (0.2)	0 (0.0)	
Parainfluenza 3	0 (0.0)	1 (0.0)	
Parainfluenza 4	0 (0.0)	3 (0.1)	
Respiratory syncytial virus	0 (0.0)	39 (1.9)	**.003**
*Mycoplasma pneumoniae*	0 (0.0)	0 (0.0)	
*Chlamydophila pneumoniae*	0 (0.0)	3 (0.1)	
*Bordetella pertussis*	0 (0.0)	1 (0.0)	
Rhinovirus/Enterovirus	8 (1.7)	172 (8.3)	**<.001**

Note. SARS-CoV-2, severe acute respiratory syndrome coronavirus.

a Statistical significance determined by both χ^2^ and Fisher exact tests; χ^2^ *P* values listed.

## Discussion

Our results suggest that infection with other respiratory pathogens is uncommon among patients with COVID-19. Notably, the median age of coinfected patients was nearly 20 years younger than those only infected with SARS-CoV-2. This observation is consistent with established literature that community-acquired viral coinfections are more common in younger populations.^4^

The difference in coinfection frequency from recently published reports may be partially explained by seasonal and geographic variability in respiratory pathogens. During the study period, the Illinois Department of Public Health noted a decline in influenza tests positivity from 14.9% to 1.6% between the weeks ending March 14, 2020, and April 11, 2020, respectively.^5^ As rates of infection due to influenza and other seasonal respiratory pathogens continue to decline into summer, coinfections among patients with COVID-19 are expected to decrease as well. Notably, differences in clinical presentation and any concurrent microbiologic data were not investigated in this brief report; further analysis of these variables may offer clarity on which patients are at highest risk for coinfection.

In the setting of limited SARS-CoV-2 testing and concerns for low sensitivity of SARS-CoV-2 RT-PCR, many hospitals utilize RP results to determine the likelihood of COVID-19 among PUIs. Although further evaluation of different institutional and regional experiences is needed to improve testing algorithms, our results support this use of RPs to risk stratify symptomatic PUIs where widespread SARS-CoV-2 testing may still not be available.
